# Identification of Disease Resistance Parents and Genome-Wide Association Mapping of Resistance in Spring Wheat

**DOI:** 10.3390/plants11212905

**Published:** 2022-10-28

**Authors:** Muhammad Iqbal, Kassa Semagn, Diego Jarquin, Harpinder Randhawa, Brent D. McCallum, Reka Howard, Reem Aboukhaddour, Izabela Ciechanowska, Klaus Strenzke, José Crossa, J. Jesus Céron-Rojas, Amidou N’Diaye, Curtis Pozniak, Dean Spaner

**Affiliations:** 1 Department of Agricultural, Food and Nutritional Science, University of Alberta, 4–10 Agriculture-Forestry Centre, Edmonton, AB T6G 2P5, Canada; 2 Agronomy Department, University of Florida, Gainesville, FL 32611, USA; 3 Lethbridge Research and Development Centre, Agriculture and Agri-Food Canada, 5403 1st Avenue South, Lethbridge, AB T1J 4B1, Canada; 4 Morden Research and Development Centre, Agriculture and Agri-Food Canada, 101 Route 100, Morden, MB R6M 1Y5, Canada; 5 Department of Statistics, University of Nebraska—Lincoln, Lincoln, NE 68583, USA; 6 Biometrics and Statistics Unit, International Maize and Wheat Improvement Center (CIMMYT), Km 45 Carretera, Veracruz 52640, Mexico; 7 Crop Development Centre and Department of Plant Sciences, University of Saskatchewan, 51 Campus Drive, Saskatoon, SK S7N 5A8, Canada

**Keywords:** association mapping, disease resistance, trait donor, prairie provinces, priority 1 diseases, restrictive linear phenotypic selection index, selection index, western Canada

## Abstract

The likelihood of success in developing modern cultivars depend on multiple factors, including the identification of suitable parents to initiate new crosses, and characterizations of genomic regions associated with target traits. The objectives of the present study were to (a) determine the best economic weights of four major wheat diseases (leaf spot, common bunt, leaf rust, and stripe rust) and grain yield for multi-trait restrictive linear phenotypic selection index (RLPSI), (b) select the top 10% cultivars and lines (hereafter referred as genotypes) with better resistance to combinations of the four diseases and acceptable grain yield as potential parents, and (c) map genomic regions associated with resistance to each disease using genome-wide association study (GWAS). A diversity panel of 196 spring wheat genotypes was evaluated for their reaction to stripe rust at eight environments, leaf rust at four environments, leaf spot at three environments, common bunt at two environments, and grain yield at five environments. The panel was genotyped with the Wheat 90K SNP array and a few KASP SNPs of which we used 23,342 markers for statistical analyses. The RLPSI analysis performed by restricting the expected genetic gain for yield displayed significant (*p* < 0.05) differences among the 3125 economic weights. Using the best four economic weights, a subset of 22 of the 196 genotypes were selected as potential parents with resistance to the four diseases and acceptable grain yield. GWAS identified 37 genomic regions, which included 12 for common bunt, 13 for leaf rust, 5 for stripe rust, and 7 for leaf spot. Each genomic region explained from 6.6 to 16.9% and together accounted for 39.4% of the stripe rust, 49.1% of the leaf spot, 94.0% of the leaf rust, and 97.9% of the common bunt phenotypic variance combined across all environments. Results from this study provide valuable information for wheat breeders selecting parental combinations for new crosses to develop improved germplasm with enhanced resistance to the four diseases as well as the physical positions of genomic regions that confer resistance, which facilitates direct comparisons for independent mapping studies in the future.

## 1. Introduction

Canada is the fifth-largest wheat producer and one of the top four wheat exporters globally, accounting for 13.9% of the global wheat market worth US$7.1 billion (https://oec.world/en/profile/hs/wheat; accessed on 8 August 2022). Continuous development and registration of high-yielding wheat cultivars with improved end-use quality traits, early maturity, tolerance to lodging, and at least intermediate resistance to five priority diseases are of paramount importance to maintaining wheat productivity in the country. The selection of genetically diverse parents by leveraging all phenotype and genomic data for simple crosses, top-crosses (three-way crosses), double-crosses, and backcrosses is the first step towards successfully developing modern cultivars, which determines the genetic variance within the population to warrant maximum selection progress [1,2]. The relevance of any cross depends on the expected performance of its superior progenies, which is a linear combination of the mean of the population and its standard deviation [3]. Different methods have been used for selecting parents, including the progeny variance prediction [4,5], optimal haploid value [6], genomic estimated breeding value [2,7], genomic usefulness criteria [1], predicted cross value [8], and a Monte Carlo simulation method [9,10].

Although the selection of parents slightly differs depending on the mating systems of the crop and the breeding method, most breeders use combinations of pedigree relationship, phenotypic performance for target traits, adaptability and yield stability, genetic distance (dissimilarity), and genetic relatedness (kinship) matrices computed from genomic data, and genotype quality control data. The phenotype data used for selecting parents could be combinations of agronomic traits, grain yield and characteristics, end-use quality traits, pre-harvest sprouting, and reaction to major diseases [11,12]. In western Canada, the Canadian Food Inspection Agency, the authority mandated for registering new cultivars, demands that candidate cultivars must display improved performance for numerous traits, including early maturity, short stature with strong straw, high grain yield, high grain protein content, improved end-use quality traits specific to each milling class, and at least intermediate resistance to five priority diseases (Fusarium head blight, common bunt, stem rust, leaf rust, and stripe rust). Leaf spot is a complex second-priority disease that has been frequently observed in the prairie provinces of Canada depending on the genetics of the cultivars, the wheat class, soil-climate conditions, and cultural practices [13,14,15].

The development of a modern cultivar that possesses all desirable traits is a complicated and resource-intensive process that can be done either by simultaneously selecting multiple traits using selection indices [16,17,18,19] or by sequentially selecting each trait at different cycles (generations) during the line development stage of breeding programs [20,21]. The Kempthorne and Nordskog restrictive linear phenotypic selection index (RLPSI) [22] is one of the multi-trait indices used for selecting parents or progeny, which has been reviewed in detail in previous studies [17,19,20,23,24,25,26,27]. The purpose of restricted (constrained) index selection is to maximize the genetic progress of some traits by leaving the expected genetic gain of others unchanged [17]. In a previous study using the RLPSI and the Smith linear phenotypic selection index [28], we reported 22 genotypes based on days to maturity (83–93 days), plant height (72–100 cm), grain yield (4.0–6.2 t ha^−1^), and grain protein content (14.6–17.7%) as possible parents for initiating new crosses towards developing improved germplasm for cultivation under low and high nitrogen management systems [29]. However, the reactions of each selected genotype to major wheat diseases have not been considered, which forms one of the bases of this study.

The economic importance of stripe rust (*P. striiformis* f. sp. *tritici*), leaf rust (*Puccinia triticina* f. sp. *tritici*), common bunt caused by both *Tilletia tritici* and *T. laevis*, and leaf spot complex caused by five fungal species (*Pyrenophora tritici-repentis*, *Phaeosphaeria nodorum*, *Mycosphaerella graminicola*, *Phaeosphaeria avenaria*, and *Cochliobolus sativus*) in Canadian wheat has been reviewed in previous studies [14,30,31]. Resistance to each disease is controlled by race-specific and race-nonspecific genes as well as quantitative trait loci (QTL). Currently, a total of 83 race-specific *Yr* genes and 80 *Lr* genes [32], 16 race-specific common bunt genes (*Bt1* to *Bt15* and *BtP*) [33], and 3 *Ptr* genes associated with insensitivity to host-specific toxins (*ToxA*, *ToxB,* and *ToxC*) [34] have been reported in wheat. In addition, a total of 31 quantitative trait loci (QTL) for common bunt, 384 QTLs for stripe rust, 557 QTLs for leaf rust, and 92 QTLs for tan spot have also been reported in wheat between 1996 and 2021 [35]; more information on this can be found at http://www.wheatqtldb.net (accessed on 8 August 2022). In a previous genome-wide study in Canadian spring wheat, we reported one genomic region associated with stripe rust resistance on chromosome 2A and tan spot on 2B, two regions for leaf rust on 2B, and three regions for common bunt on 2B, 4B, and 7A, which individually accounted for 8.7–20.9% of the phenotypic variance [36]. However, that study was based on a smaller subset of 87 spring wheat cultivars and a consensus genetic map that may have restricted our ability to identify more genomic regions. In addition, the effect of each region seems upward biased due to the small population size, and the genetic positions of the identified region seem incorrect when compared with their corresponding physical positions, which form another basis of the present study. The recently released International Wheat Genome Sequencing Consortium (IWGSC) (http://wheat-urgi.versailles.inra.fr/; accessed on 8 August 2022) provided researchers an opportunity for multiple purposes, including comparing QTL discovery results from independent studies [37,38]. Here, we used an association mapping panel to (a) assess the effect of economic weights on multi-trait selection index related to disease resistance (leaf spot, common bunt, leaf rust, and stripe rust), and grain yield using RLPSI, (b) select the top 10% genotypes with better resistance to the four diseases with at least 4.3 t ha^−1^ grain yield, and (c) map genomic regions associated with resistance to each disease using the IWGSC RefSeq v2.0 physical map.

## 2. Results

### 2.1. Phenotypic and Genetic Variation

**Figure 1:**
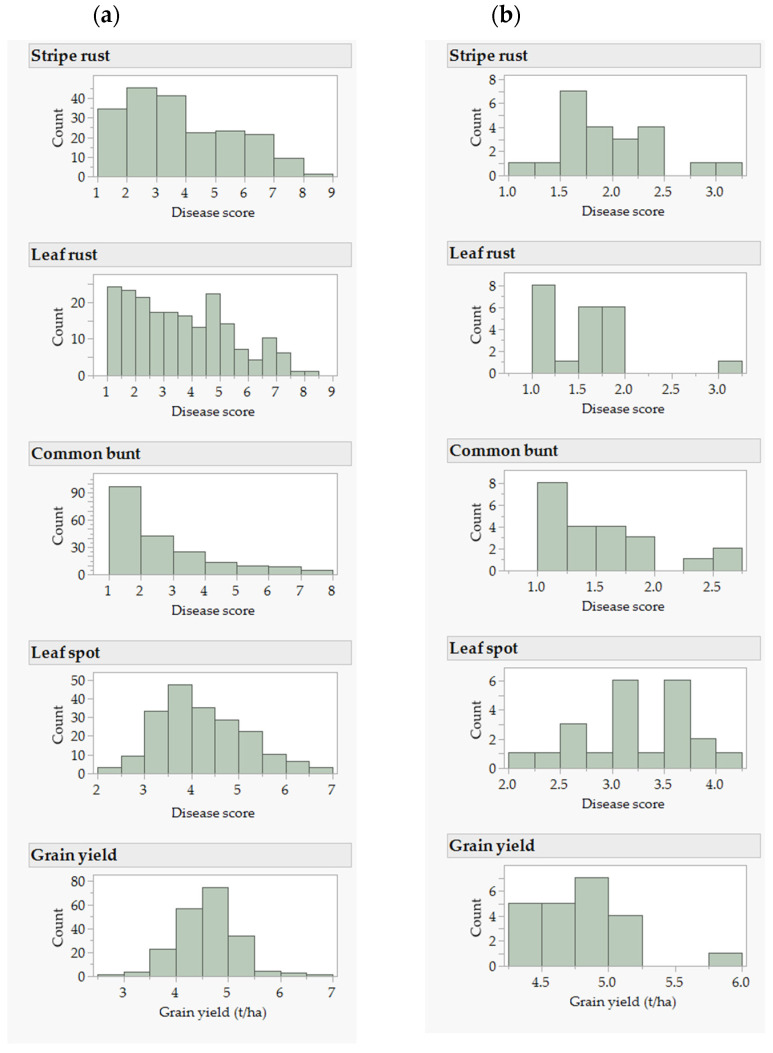
Frequency distribution of the best linear unbiased estimators (BLUEs) of disease severity and grain yield computed from all environments for (**a**) all 196 genotypes, and (**b**) the 22 selected genotypes. For all four diseases, we considered genotypes with a mean disease severity score of 1–2, 2.1–3.0, 3.1–5.0, 5.1–7.0, and 7.1–9.0 as resistant, moderately resistant, intermediate, moderately susceptible, and susceptible, respectively.

**Figure 2:**
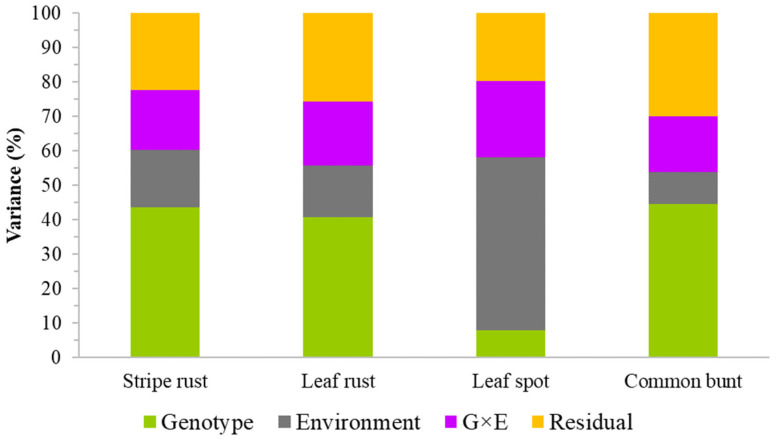
Partitioning of total variance into genotypes (G), environments (E), G × E interactions, and residual (error) variance components.

Reactions to common bunt, leaf spot, leaf rust, and stripe rust were recorded in eight, four, three, and two environments, respectively. The Pearson correlation coefficients between pairs of environments (Table S1) for stripe rust, leaf rust, and common bunt were moderate to high (0.47 ≤ r ≤ 0.94) as compared with an erratic correlation for leaf spot (−0.06 ≤ r ≤ 0.82). The best linear unbiased estimator (BLUEs) computed from all environments to represent the overall mean disease scores ranged from 2.2 to 6.8 for leaf spot, from 1.0 to 7.9 for common bunt, from 1.0 to 8.1 for leaf rust, and from 1.2 to 8.1 for stripe rust (Figure 1, Table S2). The broad-sense heritability computed from all environments was 0.22 for leaf spot, 0.69 for common bunt, 0.78 for leaf rust, and 0.89 for stripe rust. The high environmental variance (50.2%) in leaf spot had reduced broad-sense heritability, which was three to five-fold greater than the 9.1–16.7% environmental variance observed for the other three diseases (Figure 2). The genotypic variance for leaf spot (8.0%) was five to six-fold smaller than the 40.7–44.7% genotypic variance observed for the other three diseases. The genotype by environment interaction (G × E) was comparable in all four diseases, which ranged from 16.3% to 22.2% (Figure 2). The correlations among pairs of diseases computed by combining all environments ranged from 0.02 between common bunt and stripe rust to 0.56 between stripe rust and leaf rust (Figure S1) and were significant (*p* < 0.05) in all diseases except between stripe rust and common bunt (*p* = 0.76). 

As summarized in Table S3, the 23342 polymorphic SNPs were distributed across all 21 wheat chromosomes with 277–2005 markers per chromosome. Missing data per marker before and after imputation varied from 0 to 29.9% and from 0 to 1.6%, respectively. The detailed molecular diversity, genetic relatedness, and population structure of the diversity panel have been reported in our previous study [39]. Briefly, three-dimensional plots of PC1 (11.9%), PC2 (7.8%), and PC3 (5.0%) from the principal component analysis showed three groups (Figure S2), but the patterns of grouping were not clear concerning the origin of the germplasm, breeding history/periods, and milling classes. The identity-by-state (IBS) genetic distance computed among pairs of the 196 genotypes varied from 0.010 between AC Vista and CDC NRG003 (both CWSP class) to 0.491 between Oslo (CNHR) and SY087 (CWSP) with an overall mean of 0.349 (Table S4). Most of the pairs (77.6%) differed by 30–49% of the scored alleles as compared with just 3.8% of the pairs of genotypes that differed by <2% of the alleles. 

### 2.2. Comparison of Economic Weights for Multi-Trait Selection

Breeders select parents based on a single trait at a time or multiple traits simultaneously using indices. We first conducted a single trait selection by comparing the overall disease score with checks, which identified 41 genotypes with resistance (11) and moderate resistance (30) for stripe rust as compared with the Lillian and AC Andrew, respectively. For leaf rust, we selected 85 genotypes that had either the same or better level of resistance as compared with the moderately resistant McKenzie. Twenty-nine genotypes had either the same or better level of common bunt resistance as compared with McKenzie, and 12 genotypes had a moderate level of leaf spot resistance as compared with Neepawa. Of the selected genotypes based on a single disease, GP112 was selected based on all four diseases; both BYT14-11 and Muchmore were selected based on common bunt, stripe rust, and leaf rust; 21 genotypes were selected based on both leaf and stripe rust scores; 10 genotypes were selected based on both common bunt and leaf rust, and one genotype was selected based on common bunt and stripe rust (Table S2).

**Figure 3:**
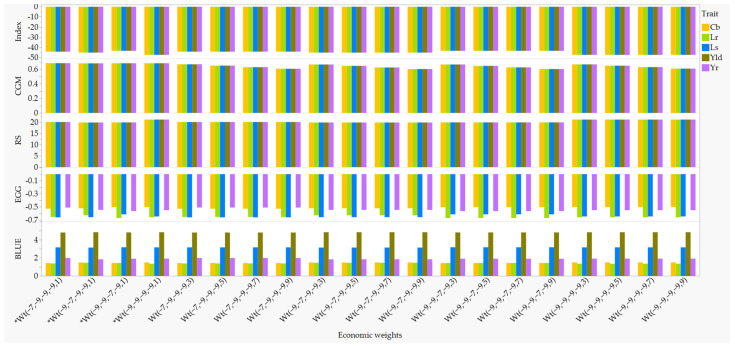
Bar graphs of best unbiased estimators (BLUEs) computed from all environments and the four selection parameters: the expected genetic gain (EGG) per trait, response to selection (RS), index, and the correlations between the index and genetic merit (CGM). The plots were made only for the top 20 of 3215 economic weights of which the four selected economic weights have * as a prefix. Trait acronyms—Cb (common bunt), Lr (leaf rust), Ls (leaf spot), Yld (grain yield), and Yr (stripe rust). See Table S5 for details.

To identify the optimal economic weights for multi-trait index-based parent selection, we first compared the index, response to selection (RS), expected genetic gain per trait (EGG), and correlation between the index and the net genetic merit (CGM) across 3125 combinations of economic weights. The weights involved common bunt, leaf spot, stripe rust, and leaf rust (each with a weight of −1, −3, −5, −7, and −9) and grain yield (1, 3, 5, 7, and 9). As summarized in Table S5A, EGG, RS, and CGM in each disease varied from −0.84 to −0.15, from 2.4 to 21.2, and from 0.19 to 0.70, respectively, while the EGG for grain yield remained unchanged. The overall BLUEs for each disease showed highly positive correlations with EGG (0.78 < r < 0.93, *p* < 0.01; Table S5B), which suggests that selection for disease resistance should be based on smaller genetic gains. We also observed highly significant negative correlations between the RS and the index (−0.96 < r < −0.94, *p* < 0.001). ANOVA with the Tukey-Kramer multiple comparisons of means revealed significantly greater (*p* < 0.01) RS and CGM, but smaller disease scores, index, and EGG when the economic weight for each disease was set to −9 or −7 (Table S5C). Smaller disease scores and EGG per trait are indicative of resistance genotypes. Grain yield was also significantly greater when the economic weights for stripe rust, leaf rust, and leaf spots were set to −9 or −7, and common bunt at −1 or −3. While the economic weights for grain yield did not show statistically significant differences in the EGG per disease and RS, CGM was significantly greater when grain yield was equal to 1. Based on the overall performance of the economic weights for the different selection parameters, we then selected 4 of the 3125 economic weights (Figure 3, Table S5D) corresponding to −7 and −9 for stripe rust, leaf rust, and leaf spot; −9 for common bunt, and 1 for grain yield as follows: Wt1 (−7, −9, −9, −9, 1), Wt2 (−9, −7, −9, −9, 1), Wt3 (−9, −9, −7, −9, 1), and Wt4 (−9, −9, −9, −9, 1).

### 2.3. Disease Resistant Parents Selected Using Single-Trait and Multi-Trait RLPSI

We selected genotypes with at least a moderate level of resistance to each of the four diseases, which identified a total of 116 genotypes. Seventy-three of them were selected based on just one of the four diseases, 36 of them were selected based on two diseases, 6 of them were selected based on three diseases, and only one was selected based on all four diseases. Such results demonstrated the challenge of simultaneously selecting genotypes with at least a moderate level of resistance for combinations of 3–4 diseases (Table S2B). To simplify the selection of possible parental genotypes for future use, we then used the multi-trait RLPSI that requires *a priori* knowledge of optimal economic weights for analyses. 

**Figure 4:**
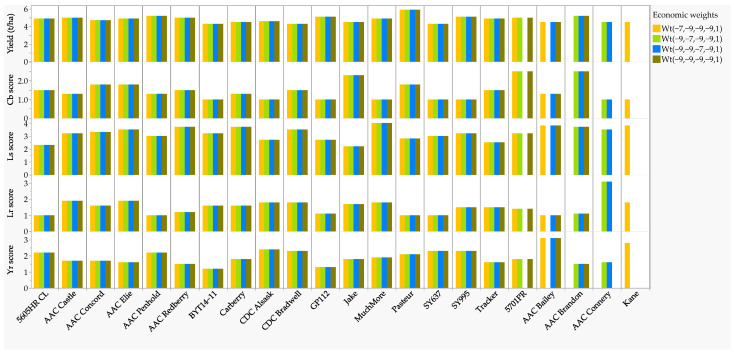
Bar graph of the overall disease scores (BLUE) of the 22 lines and cultivars chosen using 4 out of the 3125 economic weights. Of the 22 genotypes, 17 were selected using all four economic weights, Kane and AAC Connery were selected using one and two weights, respectively, and the remaining three genotypes (5701PR, AAC Bailey, and AAC Brandon) were selected using three weights. See Table S5E for details.

Using the four best economic weights identified in the previous section, we then selected a total of 22 genotypes as possible parents (trait donors) of the four wheat diseases (Table 1 and Table S5E,F, Figure 4, ) of which 17 genotypes were consistently selected using all four economic weights. The remaining five genotypes were selected using one of the RLPSI economic weights (Kane), two weights (AAC Connery), and three weights (5701PR, AAC Bailey, and AAC Brandon). The mean disease scores of the 22 selected genotypes varied from 1.2 to 3.1 for stripe rust, from 1.0 to 3.1 for leaf rust, from 1.0 to 2.5 for common bunt, and from 2.2 to 4.0 for leaf rust, and produced between 4.3 to 5.9 t ha^−1^ grain yield (Table 1). Twelve of the 22 selected genotypes were from the Canada Western Red Spring, and the remaining genotypes were from the Canada Western Special Purpose (2), the Canada Prairie Spring Red (4), and the Canada Northern Hard Red (4) market classes. Eleven of the 22 genotypes were developed by Agriculture and Agri-Food Canada (AAC Bailey, AAC Brandon, AAC Connery, AAC Elie, AAC Penhold, AAC Concord, AAC Redberry, Carberry, AAC Castle, Kane, and Muchmore); the remaining genotypes were developed and/or registered by the Syngenta Canada Inc., Calgary, AB (5605HR CL, GP112, SY637, and SY995), the University of Alberta wheat breeding program (BYT14-11, Jake, and Tracker), the University of Saskatchewan (CDC Alsask and CDC Bradwell), Nutrien AG Solutions Inc., Loveland, CO (5701PR), and Plantomar Ltd (Pasteur). 

When genotypes selected based on the multi-trait RLPSI were compared with those selected based on single-trait, GP112 was selected not only based on the RLPSI, but also the single-trait analyses performed in all four diseases. CDC Alsask, Muchmore, BYT14-11, Jake, and Tracker were selected based on RLPSI and three of the four diseases in the single-trait analyses. Thirteen of the 22 cultivars (5605HR CL, 5701PR, AAC Brandon, AAC Connery, AAC Elie, AAC Concord, AAC Redberry, Carberry, AAC Castle, Kane, Pasteur, SY637, and SY995) were selected based on the RLPSI and two of the four diseases from the single-trait analyses. The remaining three genotypes (AAC Bailey, AAC Penhold, and CDC Bradwell) were selected based on the RLPSI and only leaf rust from single-trait analysis (Table 1, Table S2B). 

### 2.4. Genome-Wide Association Mapping

**Figure 5:**
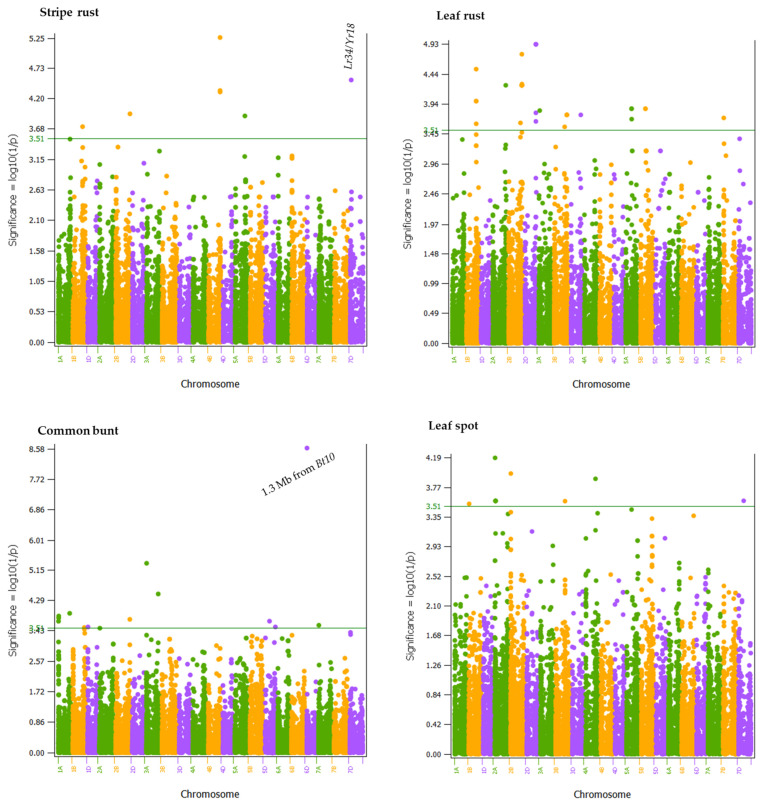
Manhattan plots of Log_10_(1/p) values computed using weighted mixed linear model, PC1 to PC3 from principal component analysis to account for population structure, best linear unbiased estimators (BLUEs) of disease scores computed from all environments, and genotype data of 23,342 polymorphic SNPs. The horizontal line shows the threshold *p*-value of 3.1 × 10^−4^ (3.51). The A, B, and D genomes are in green, orange, and purple colors, respectively. Chromosomes and physical positions are shown on the *x*-axis. See Table S6 for detailed results.

A weighted mixed linear model analysis conducted using BLUEs computed from disease scores in the individual and combined environments identified a total of 560 SNPs significantly associated with the four diseases (Table S5B) of which 66 SNPs were significantly associated in the combined disease scores of all environments (Table S6C). The latter included 13 SNPs for common bunt, 34 SNPs for leaf rust, 7 SNPs for stripe rust, and 12 SNPs for leaf spot. In the interest of brevity, we have only presented the results of the 66 SNPs identified in the combined disease scores of all environments, which were located at 37 genomic regions in all chromosomes except 4D, 6A, and 6B (Table 2, Figure 5, Table S5D). There were 13 SNPs significantly associated with common bunt resistance, which were located at 12 regions on chromosomes 1A (4.4 Mb, 13.4–14.0 Mb, and 556.9 Mb), 1B (645.3 Mb), 1D (10.7 Mb), 2B (811.0 Mb), 3A (10.3 and 671.3 Mb), 5D (244.1 and 565.9 Mb), 6D (7.4 Mb), and 7A (15.8 Mb). Each genomic region accounted for 6.6–19.6% and together explained 97.9% of the total phenotypic variance of common bunt in the combined environments (Table 2). The 34 SNPs associated with leaf rust resistance were located at 13 genomic regions on chromosomes 1B (547.3–549.6 Mb), 2A (758.3 Mb), 2B (690.9 and 771.9–778.2 Mb), 2D (624.6–625.0 Mb), 3A (51.6 Mb), 3B (616.1 and 743.9 Mb), 3D (550.3 Mb), 5A (331.8 and 338.7 Mb), 5B (281.2–284.7 Mb), and 7B (36.2 Mb). Each genomic region accounted for 6.6–8.3% and together accounted for 94.0% of the total phenotypic variance of the leaf rust reaction recorded in all environments.

The 7 SNPs associated with stripe rust resistance were located at 5 genomic regions on chromosomes 1B (540.6 Mb), 2B (812.2 Mb), 4B (638.8 Mb), 5A (547.6 Mb), and 7D (49.0 Mb), which individually accounted for 7.0–8.9% and together for 39.4% of the total phenotypic variance of the stripe rust severity at eight combined environments. The 12 SNPs associated with leaf spot resistance were located across seven genomic regions on chromosomes 1B (18.2 Mb), 2A (19.0 and 45.9 Mb), 2B (19.5 Mb), 3B (557.8 Mb), 4A (580.8 Mb), and 7D (266.7 Mb). Each of these regions individually accounted for 6.6–7.9%, and together for 49.1% of the total phenotypic variance of the leaf spot severity in three combined environments (Table 2).

**Figure 6:**
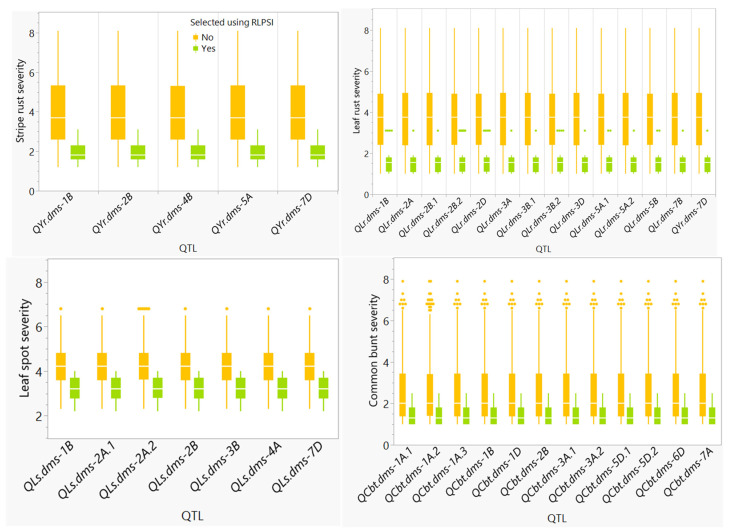
Comparisons of the overall disease severity recorded in all combined environments between selected and unselected genotypes at each genomic region identified using genome-wide association analysis. Disease resistant donor genotypes were selected based on a restricted linear phenotypic selection index. The dots indicate outlier scores.

Figure 6 and Table S7 compared the reaction of the 22 genotypes selected based on the multi-trait RLPSI with the remaining 174 unselected genotypes at each of the 37 QTL identified using GWAS. In all 37 pairs of comparisons, the selected genotypes had significantly (*p* < 0.01) smaller disease severity than the unselected genotypes that displayed highly erratic reactions in all four diseases. The identified QTLs likely contributed the observed resistance among most selected genotypes. 

## 3. Discussion

The usefulness of a cross can be assessed based on its expected genetic gain per trait, which depends on the genetic variation of the trait of interest in the breeding material, the heritability of the trait under selection, selection intensity, and the time required to complete a breeding cycle or generation interval [7,40]. Our data revealed the presence of large molecular and phenotypic variations in all four diseases. However, broad-sense heritability for leaf spot severity was quite low (0.22) as compared with the other three diseases (0.69–0.89), which was also evident from the low genotypic and high environmental variances (Figure 2). Our results agree with previous studies that reported highly variable heritability for leaf spot (0.21–0.99) in diverse spring wheat populations [41,42] depending on the genetic backgrounds, environments, the fungal species involved, and mixture of isolates. Economic weights affect the ranking of the selected genotypes, the index, the correlation between the index and the net genetic merit, the expected genetic gain per trait, and the response to selection [16,23,43,44]. We selected genotypes with the lowest disease scores, expected genetic gain per trait, and lowest index but the highest response to selection and correlation between the next genetic merit and index (Figure 3) plus with at least 4.3 t ha^−1^ grain yield. Of the 22 genotypes, five of those selected in the present study based on disease resistance and grain yield were also previously selected based on their superior performance for agronomic traits under contrasting nitrogen management systems [29], which included AAC Brandon, AAC Penhold, CDC Alsask, 5605HR CL, and AAC Castle (Table S2).

In the present study using the IWGSC RefSeq v2.0 physical map, we identified five genomic regions associated with the overall stripe rust resistance at 540.6 Mb on 1B (Tdurum_contig16865_174), at both 812.2 Mb (BS00086533_51) on 2B, at 638.8 Mb (wsnp_BE499546B_Ta_2_1, RAC875_c36213_352, and Excalibur_c49297_159) on 4B, at 547.6 Mb (Excalibur_c11045_236) on 5A, and at 49.0 Mb (wMAS000004) on 7D (Figure 5, Table S6D). Previous mapping studies conducted in diverse types of biparental populations and association mapping panels reported numerous genes and QTLs that confer resistance to stripe rust on all wheat chromosomes, including 1B (33 QTLs), 2B (48 QTLs), 4B (19 QTLs), 5A (15 QTLs), and 7D (22 QTLs) [35]. Recently, for example, Aoun et al. [45] reported the physical positions of 56 QTLs that confer stripe rust resistance in a global durum wheat collection, including four on chromosomes 1B, one on 2B, three on 4B, and five on 5A; however, none of these QTLs were located close to the QTLs identified in the present study. The long arm of chromosome 2B harbors several race-specific stripe rust resistance genes, including *Yr5, Yr7*, *Yr43*, *Yr44*, *Yr53*, and *Yr72* [46]. *Yr5* is flanked by IWA6121 (wsnp_JD_c6010_7167084) and IWA4096 (wsnp_Ex_c5123_9088111) SNPs [47], which are physically located between 692.5–705.5 Mb in the IWGSC RefSeq v1.1 and v2.0 physical maps. In the current study, the stripe rust resistance QTL on 2B was located at 794.1–812.2 Mb in the two versions of physical maps (Table S6), which is at least 89 Mb away from the position of the *Yr5* gene. The physical position of the *Yr7* varied from 633.4 to 721.2 Mb [48] depending on the methods, which overlaps with the *QLr.dms-2B.1* identified in the current study (Table S6). *Yr50* [49], *Yr62* [50], and *Yr68* are the three stripe rust resistance genes reported on chromosome 4BL. The wMAS000004 marker is one of the KASP causal markers designed to introgress the slow-rusting *Lr34* gene that provide durable and non-race specific resistance both at the seedling and adult plant stages [51]. The wMAS000004 marker passed the significant threshold value in three individual environments and the overall mean stripe rust disease score and accounted for 8.6–11.3% of the phenotypic variance of all combined environments (Table S6B). For leaf rust, wMAS000004 was detected in one of the four environments but not in all combined environments. A comparison of the effect of the 68 lines and cultivars that had the CC genotype at the wMAS000004 marker showed on average smaller leaf spot, leaf rust, and stripe rust severity than the 128 lines and cultivars with the TT genotype regardless of the environments (Figure S4). As reviewed by Lagudah et al. [52], the region that harbors *Lr34* also coincides with several other disease resistance genes, including stripe rust (*Yr18*), powdery mildew (*Pm38*), leaf tip necrosis (*Ltn1*), and barley yellow dwarf virus (*Bdv1*) genes. Since the *Lr34* gene has been reported to interact with other resistance genes to increase the level of resistance [53,54,55,56], it has been extensively introgressed in the Canadian wheat cultivars [57,58]. It should, however, be noted that the exact position of the *Lr34* gene on chromosome 7D differs by ~1.5 Mb depending on the version of the physical maps (7D:47412062-47424490 in RefSeq v1.0 versus 7D:48955909-48955803 in RefSeq v2.0). A candidate gene search in Ensemble Plants by merging these two positions (7D: 47412062-48955803) identified 17 *Triticum aestivum* genes, including *Lr34* (*TraesCS7D02G080300*), Cytochrome P450 (*TraesCS7D02G080400* and *TraesCS7D02G080700*), *Lectin receptor kinase 1* (*TraesCS7D02G080500*), *Lectin receptor kinase 2* (*TraesCS7D02G080600*), and *Carboxypeptidase SOL1* (*TraesCS7D02G081800*) (Table S6E).

For leaf rust, we identified 13 genomic regions across eleven chromosomes (1A, 2A, 2B, 2D, 3A, 3B, 3D, 5A, 5B, and 7B), which individually accounted for 6.6–8.3% of the phenotypic variance of all four environments (Table S6D). Three of the SNPs located on chromosome 2B at both 690.9 Mb (BobWhite_c18540_351) and 771.9–778.12 Mb (BS00079941_51), and another SNP on 2D at 624.6–625.0 Mb (RAC875_c52856_250) were also previously found associated with leaf rust resistance in 81 Canadian spring wheat cultivars evaluated at four environments at the University of Alberta South Campus [36]. Each region in the previous study, however, individually accounted for 18.1–19.2% of the phenotypic variance of the overall leaf rust resistance across all environments, which was over two-fold greater than the effect in the present study. Chromosomes 2B harbors nine leaf rust resistance genes, including *Lr13*, *Lr16*, *Lr23*, *Lr35*, *Lr48*, *Lr50*, *Lr58*, *Lr73*, and *Lr82*, while 2D harbors another six leaf rust resistance genes (*Lr2a-c*, *Lr15*, *Lr22a-b*, *Lr39*, *Lr54*, and *Lr80*). 

*Lr16* is located on 2BS and contributes partial resistance to leaf rust at seedling stage alone or together with *Lr34* in the Canada wheat germplasm [59,60]. The six SNP markers that co-segregated with the *Lr16* in four RIL wheat populations [61] were mapped between 6.2 Mb and 74.8 Mb. Recently, McCallum and Hiebert [54] used one of those SNPs (2BS-5203447_kwm742) to differentiate doubled haploid lines with and without the *Lr16* resistance alleles, which is located at 62.2 Mb and 69.7 Mb in the IWGSC RefSeq v1.0 and v2.0 physical maps, respectively. The *QLr.dms-2B.1* at 690.9 Mb and *QLr.dms-2B.2* at 771.9–778.2 Mb associated with leaf rust resistance were located on the long arm of 2B and far from the *Lr16* gene. In addition to the race-specific leaf rust resistance genes reported in wheat [32], at least 370 leaf rust QTLs have also been reported on 11 chromosomes [35], which are available at the WheatQTLdb (http://www.wheatqtldb.net/fungal_new.php; accessed on 8 August 2022). *Lr2a*, a seedling leaf rust resistant gene, is located on chromosome 2DS, which was found co-segregating with kwh740 (Excalibur_c1944_1017) in two Canadian wheat mapping populations (Superb/BW278 and Superb/86ISMN 2137) that shared Superb as a common parent [62]. Excalibur_c1944_1017 is physically located at 61.2 Mb and 63.7 Mb in the IWGSC RefSeq v1.0 and v2.0 maps, respectively. The *QLr.dms-2D* QTL uncovered in the present study was, however, located on the long arm at 621.8–763.6 Mb and 624.6–624.9 Mb in the IWGSC RefSeq v1.0 and v2.0 maps, respectively (Table S6).

D_contig17056_55 showed the strongest association with common bunt resistance in the individual and combined environments, which was mapped on the short arm of chromosome 6D (6D:6966722-7431984) depending on the physical map. This region accounted for 16.9% of the phenotypic variance of common bunt reaction in the combined environments and 8.0–12.4% in the individual environments (Table S6). A total of 16 protein coding genes are located within this physical interval (Table S6E), including P-loop containing nucleoside triphosphate hydrolases superfamily protein (*TraesCS6D02G017000*). Both the *Bt9* and *Bt10* genes are located on the long and short arms of chromosome 6D, respectively [63,64]. *Bt10* has been effective against *Tilletia tritici* and *T. laevis* races known in western Canada and has been extensively used in spring wheat breeding programs in the region [65]. To get the physical position of the *Bt10* gene, which seem near the genomic region identified in the present study, we blasted the sequences of the forward primer [GTTTTATCTTTTTATTTC (FSD)] reported by Laroche et al. [66] on the Gramene database, which aligned at 6D:5703192-5720941. The position of the *Bt10* marker was about 1.3 Mb away from the *QCbt.dms-6D* identified in this study.

The chromosomal distribution of all disease resistance QTLs identified in the present study varied from one on each of the nine chromosomes (1D, 2D, 3D, 4A, 4B, 5B, 6D, 7A, and 7B) to five on 2B (Table 2). Previous studies have reported multiple genes and QTLs associated with resistance to different wheat diseases, including the *SrWeb*, *Sr28*, *Sr32*, *Sr39 Sr36*, *Sr40*, and *Sr47* [67], *Sr9h, Sr16* [59,68,69,70], a major effect stem rust QTL [71], and two moderate effect stem rust QTLs [66], but the physical positions of all those genes and QTLs are different from ours. The three main challenges in comparing gene and QTL discovery results among independent studies include (1) the low correlation between genetic and physical maps, (2) the lack of physical information for most markers linked with genes and QTLs that serve as a reference for comparing independent studies, and (3) the continuous improvement in the physical map information that results in disagreements on both chromosomal location and physical positions of the genes and QTLs. Gene annotation databases, such as the Ensembl Plants (https://plants.ensembl.org/index.html; accessed on 8 August 2022), are based on the IWGSC RefSeq v1.0, which partly disagrees with the improved IWGSC RefSeq v2.0 or v2.1 (http://wheat-urgi.versailles.inra.fr/; accessed on 8 August 2022). For example, the chromosomal distribution of 14 of the 66 SNPs (~21% of the markers) associated with the overall disease scores of common bunt, stripe rust, leaf spot, and leaf rust differed between the old and recent physical maps (Table S6D). The remaining 79% of the markers (52 of 66 SNPs) were mapped on the same chromosome in both versions of the physical maps, but their physical positions had shifted by 0.4–18.2 Mb with an average of 5.7 Mb per marker (Table S6A). Such discrepancies demonstrate the challenges in reliably comparing independent studies depending on the version of the physical map, which could be much more prone to error in species with large and complex genomes like wheat [71]. 

In a previous study [36], we evaluated 81 Canadian spring wheat cultivars for reactions to diseases from four to eight environments, genotyped them with 19,933 polymorphic markers, and identified 17 markers significantly associated with common bunt (10 SNPs), leaf rust (5 SNPs), leaf spot (1), and stripe rust (1 SNP each), which individually explained 8.7–21.0% (17.0% on average) of the phenotypic variance in the combined environments. The 66 significant MTAs identified in the present study were nearly 4-fold greater than those identified in the previous study, while the mean phenotypic variance explained by each MTA decreased by half (range: 6.6–16.9%, mean 7.6%). The Beavis effect is common problem in smaller population sizes that restricts the ability to detect QTLs that account for a smaller proportion of the phenotypic variance, while it overestimates the effect of moderate to major effect QTLs [72,73,74]. Differences in marker density and trait heritability may have also partly contributed to the number and effects of QTLs detected in the two studies [75,76]. The number of environments for both stripe rust and leaf rust in both studies was the same, while common bunt and leaf spot had two more environments in the previous study.

## 4. Materials and Methods

### 4.1. Genotyping and Phenotyping

We used an association mapping panel that consisted of 176 spring wheat cultivars registered from 1905 to 2018, and 20 advanced breeding lines that have not been registered (Table S2). For simplicity, we used genotypes to refer to both advanced breeding lines and cultivars. The panel originated from 15 breeding programs (institutions) and represented the eight milling classes (https://www.grainscanada.gc.ca/en/grain-quality/grain-grading/wheat-classes.html; accessed on 8 August 2022). The cultivars and lines were genotyped with wheat 90K iSelect array at the University of Saskatchewan, Saskatoon, Canada, and with a few Kompetitive Allele-Specific PCR (KASP) markers using the Biosearch Technologies (https://www.biosearchtech.com/; accessed on 8 August 2022) service lab, Beverly, MA, USA, as described in a previous study [39]. A total of 23,342 polymorphic markers were retained for statistical analysis after removing markers with a minor allele frequency of <5%, missing data of >30%, and no physical information based on the IWGSC RefSeq v2.0. The final genotype data was imputed using LinkImpute [77] implemented in TASSEL v5.2.84 [78]. The IWGSC RefSeq v2.0 information for each marker was obtained from the Wheat@URGI portal as described in our previous study [79].

The detailed methodologies for evaluating the panel for reaction to common bunt, leaf spot, stripe rust, and leaf rust, and the disease data analyses have also been described in detail in our previous open access paper [80]. Briefly, the panel and checks (AC Barrie—susceptible, CDC Imagine—intermediate, AC Andrew—moderately resistant, and Lillian—resistant) were evaluated for reaction to stripe rust in eight environments at the University of Alberta South Campus in Edmonton, Alberta, at the Lethbridge Research and Development Centre in Alberta, and near Creston in British Columbia. The panel and checks (AC Barrie—moderately susceptible to susceptible, Glenlea—intermediate, and McKenzie—moderately resistant) were also evaluated for reaction to leaf rust four times at the Morden Research and Development Centre in Manitoba, and at the University of Alberta South Campus. Stripe rust evaluation conducted near Creston was based on natural infection. In all other sites, both stripe rust and leaf rust infection were initiated by spraying spreader rows of susceptible checks with the prevalent multi-race mixture of urediniospores collected in a previous cropping season as described in another study [81]. The panel and checks (AC Domain—susceptible, AC Vista and AC Crystal—intermediate, and Neepawa—moderately resistant) were evaluated for reaction to leaf spot three times at the University of Alberta South Campus. Leaf spot infection was initiated by spraying spreader rows of susceptible checks with an equal mixture of AB7-2 and AB50-2 isolates that contain the *ToxA* gene [82] and belong to race 1 of *P. tritici-repentis* (*Ptr*). Both AB7-2 and AB50-2 isolates cause the tan spot (*Pyrenophora tritici-repentis*), but other pathogens, such as *Phaeosphaeria nodorum*, *Mycosphaerella graminicola*, *Phaeosphaeria avenaria*, and *Cochliobolus sativus* [83] display similar symptoms as tan spot, which are difficult to visually distinguish without laboratory analysis [84]. For that reason, we rated the disease severity as leaf spot. The panel and checks (Laura—and Fielder susceptible, Neepawa—intermediate, and McKenzie and AC Foremost—resistant) were also evaluated for common bunt reaction twice at the University of Alberta South Campus by treating seeds of each cultivar and line with an equal mixture of spores of L-16 of *T. laevis* and T-19 of *T. tritici* races as described in a previous study [85].

For all four diseases, severity was recorded on a scale of 0 (no visible sign or symptom = resistant) to 9 (leaf area covered with spores = highly susceptible) at some locations and from 0% to 100% at other locations. To get the same disease ratings in all locations for statistical analyses, we converted the 0–100% in to the 0 to 9 scale as follows: 0 = no infection, 1 = ≤10%, 2 = 11–20%, 3 = 21–30%, 4 = 31–40%, 5 = 41–50%, 6 = 51–60%, 7 = 61–70%, 8 = 71–80%, 9 = ≥81% of the leaf area covered by pustules or lesions. Grain yield was evaluated during the cropping seasons for five years (2017–2021) under a conventional management system at the University of Alberta South Campus using a randomized incomplete block design with two replications [29].

### 4.2. Statistical Analyses

We used Multi Environment Trial Analysis with R (META-R) v.6.04 [86] to compute best linear unbiased estimators (BLUE), variance component analyses, and broad-sense heritability by combining disease severity scores and grain yield recorded in all environments. The RLPSI analysis was performed in R for Windows x64 v3.6.1 using the code in Table S8 and three input files—(a) the overall BLUEs of each trait computed by combining all environments, (b) phenotypic and genotypic covariate matrices computed from the combined phenotype data of each trait. We first compared the effect of a total of 3125 economic weights (Table S5) on four selection parameters (EGG per trait, RS, CGM, and the index). The weights were obtained using exponential combinations of the five traits, each with five weights (−1, −3, −5, −7, and −9 for each disease and 1, 3, 5, 7, and 9 for grain yield). The analyses were performed by restricting the EGG for grain yield equal to zero (i.e., no change in grain yield potential) while the EGG of the four diseases decrease or increase without restriction. Analysis of variance (ANOVA) with Tukey-Kramer multiple mean comparisons was used to compare pairwise differences among economic weights. The top 10% of the 196 genotypes were then chosen to serve as potential parents for new crossings in the future based on the four best economic weights that had both the smallest index and EGG, but the largest RS and CGM. ANOVA, Pearson correlations, coefficient of determination, frequency distributions, and bar graphs were constructed in JMP statistical discovery software [87] v16.

Genomic regions associated with resistance to the four diseases were identified using the weighted mixed linear model in TASSEL v5.2.84. The analyses were done using the imputed SNP genotype data, the kinship matrix to account for relatedness, the first three PCs from principal components as covariates to account for population structure, and the disease severity scores (BLUEs) computed per environment and combined across all environments. The threshold for declaring significant marker-trait association was set to *p* < 3.1 × 10^−4^ or Log_10_ (1/p) value ≥ 3.5, which is the same as the threshold used in a previous study [88]. Genome-wide *p* values were visualized in Manhattan plots using SNPevg [89].

## 5. Conclusions

The 196 wheat genotypes showed very large phenotypic variation for reaction to leaf rust, stripe rust, and common bunt, but less so for the leaf spot. Leaf spot showed very large environmental variance with greater genotype by environment interactions and very low broad-sense heritability compared to the other three diseases. Genome-wide association mapping identified a total of 66 SNPs significantly associated with resistance to the four diseases evaluated in all combined environments, which were distributed across 37 genomic regions. Some of the regions were detected in previous studies, including one genomic region near the *Lr34* gene. Some of the detected regions are likely novel, which provides additional information to wheat researchers who aim to search for new sources of resistance and markers. Others may be known resistance genes or previously identified QTL, but the direct comparison was quite difficult due to inconsistencies in the genetic and physical maps. Single trait phenotypic selection identified a total of 116 genotypes with at least a moderate level of resistance to one or more diseases of which 22 genotypes were also selected using the multi-trait RLPSI as the best parents for initiating new crosses. The selected genotypes would be highly valuable sources of disease resistance to develop improved germplasm with enhanced resistance to the four wheat diseases without compromising grain yield. The 22 selected cultivars and lines represented CWRS (12 cultivars), CNHR (4), CPSR (4), and CWSP (2) wheat classes. Overall, the results presented here could provide wheat researchers with better knowledge in facilitating parental selections and utilizing genomic information in disease resistance breeding.

## Figures and Tables

**Table 1:** Summary of the pedigree and phenotype data of 20 cultivars and 2 unregistered lines chosen as potential parents based on a restricted linear phenotypic selection index. The last column provides additional information on whether the 22 genotypes were also chosen based on the mean severity of each disease separately.

Genotype	Pedigree	Wheat Class *	Year **	Stripe Rust (Yr)	Leaf Rust (Lr)	Common Bunt (Cb)	Leaf Spot (Ls)	Yield (t ha^−1^)	Selected Based on Each Disease
5605HR CL	99S2232-10/99S3228-4	CNHR	2013	2.2	1.0	1.5	2.3	4.9	Both Lr and Ls
5701PR	N89-3004/N87-0446//Oslo	CPSR	2002	1.8	1.4	2.5	3.2	5.0	Both Yr and Lr
AAC Bailey	9505-LP03A/Journey//Lillian	CWRS	2012	3.1	1.0	1.3	3.8	4.5	Only Lr
AAC Brandon	Superb/CDC Osler//ND744	CWRS	2013	1.5	1.1	2.5	3.7	5.2	Both Yr and Lr
AAC Connery	Somerset/BW865	CWRS	2015	1.6	3.1	1.0	3.5	4.5	Both Yr and Cb
AAC Elie	Superb/CDC Osler//ND744	CWRS	2013	1.6	1.9	1.8	3.5	4.9	Both Yr and Lr
AAC Penhold	5700PR/HY644-BE//HY469	CPSR	2014	2.2	1.0	1.3	3.0	5.2	Only Lr
AAC Concord	Lillian/Journey//9505-LP03A	CNHR	2016	1.7	1.6	1.8	3.3	4.7	Both Yr and Lr
AAC Redberry	Stettler/Glenn	CWRS	2016	1.5	1.2	1.5	3.7	5.0	Both Yr and Lr
BYT14-11	Peace/Carberry	CWRS	Unregistered	1.2	1.6	1.0	3.2	4.3	Yr, Lr, and Cb
Carberry	Alsen/Superb	CWRS	2009	1.8	1.6	1.3	3.7	4.5	Both Yr and Lr
CDC Alsask	AC Elsa/AC Cora	CWRS	2005	2.4	1.8	1.0	2.7	4.6	Lr, Cb, and Ls
CDC Bradwell	5602HR/W02330	CWRS	2015	2.3	1.8	1.5	3.5	4.3	Only Lr
GP112	99S3148-1/00S3075-3-13 ***	CWSP	Unregistered	1.3	1.1	1.0	2.7	5.1	Yr, Lr, Cb, and Ls
AAC Castle	Conquer/CDN Bison//5701PR	CPSR	2018	1.7	1.9	1.3	3.2	5.0	Both Yr and Lr
Kane	AC Domain/McKenzie	CNHR	2006	2.8	1.8	1.0	3.8	4.5	Both Lr and Cb
Muchmore	Alsen/Superb	CNHR	2009	1.9	1.8	1.0	4.0	4.9	Yr, Lr, and Cb
Pasteur	Cadenza/(Palermo/KS91WGRC11)	CWSP	2011	2.1	1.0	1.8	2.8	5.9	Both Lr and Ls
Jake	McKenzie/Alsen//BW297	CWRS	2018	1.8	1.7	2.3	2.2	4.5	Yr, Lr, and Ls
Tracker	Peace/CDC Stanley	CWRS	2018	1.6	1.5	1.5	2.5	4.9	Yr, Lr, and Ls
SY637	BW337/AC ELSA	CWRS	2016	2.3	1.0	1.0	3.0	4.3	Both Lr and Cb
SY995	99S3144-7/5701PR ****	CPSR	2014	2.3	1.5	1.0	3.2	5.1	Both Lr and Cb

* Wheat market classes: Canada Northern Hard Red (CNHR); Canada Prairie Spring Red (CPSR); Canada Western Red Spring (CWRS); Canada Western Special Purpose (CWSP). ** Year of registration or development. *** Parent 99S3148-1 has the pedigree, “N98-3049ES//AC VISTA/SUN299A”, and parent 00S3075-3-13 is “N99-3091//PIO2552/N98-3051ES”. **** The Female parent “99S3144-7” has the pedigree, “N98-3020/5700PR”, whereas “N98-3020” has the pedigree, “HY612/N91-3050//N92-3041”.

**Table 2:** Summary of the 37 genomic regions associated with stripe rust (Yr), leaf rust (Lr), leaf spot (Ls), and common bunt (Cb) resistance recorded in all combined environments. The chromosome (Chr) and physical position are based on the International Wheat Genome Sequencing Consortium (IWGSC) RefSeq v2.0. See Table S6 for details.

Region	No. of Significant SNPs	Chr	Min Position (bp)	Max Position (bp)	Phenotypic Variance (%)			
					Cb	Lr	Ls	Yr
*QCbt.dms-1A.1*	1	1A	4,383,342	4,383,342	6.9			
*QCbt.dms-1A.2*	2	1A	13,371,025	14,027,876	7.2			
*QCbt.dms-1A.3*	1	1A	556,873,095	556,873,095	7.4			
*QLs.dms-1B*	1	1B	18,203,933	18,203,933			6.6	
*QYr.dms-1B*	1	1B	540,583,488	540,583,488				7.0
*QLr.dms-1B*	4	1B	547,332,148	549,647,134		7.5		
*QCbt.dms-1B*	1	1B	645,251,547	645,251,547	6.6			
*QCbt.dms-1D*	1	1D	10,669,243	10,669,243	6.6			
*QLs.dms-2A.1*	1	2A	19,019,241	19,019,241			7.9	
*QLs.dms-2A.2*	6	2A	45,928,136	45,932,241			6.7	
*QLr.dms-2A*	1	2A	758,316,082	758,316,082		7.9		
*QLs.dms-2B*	1	2B	19,530,481	19,530,481			7.4	
*QLr.dms-2B.1*	1	2B	690,898,021	690,898,021		6.7		
*QLr.dms-2B.2*	10	2B	771,850,360	778,230,186		8.0		
*QCbt.dms-2B*	1	2B	811,019,075	811,019,075	7.1			
*QYr.dms-2B*	1	2B	812,244,914	812,244,914				7.4
*QLr.dms-2D*	5	2D	624,625,220	624,952,858		8.3		
*QCbt.dms-3A.1*	1	3A	10,279,544	10,279,544	10.4			
*QCbt.dms-3A.2*	1	3A	671,290,927	671,290,927	8.5			
*QLr.dms-3A*	1	3A	51,644,908	51,644,908		7.1		
*QLs.dms-3B*	1	3B	557,835,101	557,835,101			6.6	
*QLr.dms-3B.1*	1	3B	616,148,037	616,148,037		6.6		
*QLr.dms-3B.2*	4	3B	743,632,624	743,922,021		7.0		
*QLr.dms-3D*	1	3D	550,280,985	550,280,985		7.0		
*QLs.dms-4A*	1	4A	580,845,356	580,845,356			7.3	
*QYr.dms-4B*	3	4B	638,809,518	638,813,440				8.9
*QLr.dms-5A.1*	2	5A	331,454,313	331,884,318		7.2		
*QLr.dms-5A.2*	1	5A	338,666,459	338,666,459		6.8		
*QYr.dms-5A*	1	5A	547,615,657	547,615,657				7.4
*QLr.dms-5B*	2	5B	281,176,164	284,717,655		7.2		
*QCbt.dms-5D.1*	1	5D	244,100,829	244,100,829	7.0			
*QCbt.dms-5D.2*	1	5D	565,867,455	565,867,455	6.6			
*QCbt.dms-6D*	1	6D	7,431,984	7,431,984	16.9			
*QCbt.dms-7A*	1	7A	15,774,259	15,774,259	6.7			
*QLr.dms-7B*	1	7B	36,162,027	36,162,027		6.8		
*QYr.dms-7D*	1	7D	48,955,909	48,955,909				8.6
*QLs.dms-7D*	1	7D	266,720,824	266,720,824			6.6	

## Data Availability

All relevant files are included in this article and its Supplementary Files.

## Supplementary Materials

The following supporting information can be downloaded at: https://www.mdpi.com/article/10.3390/plants11212905/s1, Figure S1: Scatter plots of disease scores of 196 cultivars and lines based on best linear unbiased estimators (BLUEs) computed from all environments; Figure S2: A plot of the first three principal components of 196 spring wheat cultivars and lines genotyped with 23,342 polymorphic markers; Figure S3: Identity by state-based genetic distance between pair of 196 spring wheat cultivars and lines using 23,342 polymorphic markers; Figure S4: Box plots of common bunt, leaf rust, stripe rust, and leaf spot scores based on combined environments to compare the allelic effects of wMAS000004 (a KASP SNP for *Lr34*) and D_contig17056_55 that map 1.3 Mb away from the *Bt10* gene; Table S1: Pearson correlation coefficients between pairs of environments used to evaluating reaction to stripe rust, leaf rust, leaf spot, and common bunt; Table S2: Summary of the 196 spring wheat cultivars and lines used in the present study, including the best linear unbiased estimators (BLUEs) computed by combining phenotype data of all environments; Table S3: Physical information, minor allele frequency, and proportion of missing data of the 23,342 polymorphic markers used in the present study; Table S4: Pairwise genetic distance between pairs of 196 genotypes based on 23,342 imputed and polymorphic SNPs; Table S5: Summary of the Kempthorne and Nordskog Restrictive Linear Phenotypic Selection Index (RLPSI) based on five phenotypic traits and 3125 combinations of economic weights; Table S6: Significant marker trait associations identified based on disease scores recorded in individual and combined environments and weighted mixed linear model; Table S7: Analysis of Variance (ANOVA) comparing disease severity of selected and unselected genotypes at each of the 37 QTLs identified using genome-wide association analysis; Table S8: The Kempthorne and Nordskog Restrictive Linear Phenotypic Selection Index (RLPSI) code in R to compute the different selection parameters.
