# Supplementary material for: Identification of Disease Resistance Parents and Genome-Wide Association Mapping of Resistance in Spring Wheat
Source: Plants (Basel). 2022 Oct 28;11(21):2905. doi: 10.3390/plants11212905 (PMC9658635; doi:10.3390/plants11212905)
Supplement: Supplementary file 1 [file plants-11-02905-s001.zip › Figure S1-S4 R1.pptx]

## Slide 1
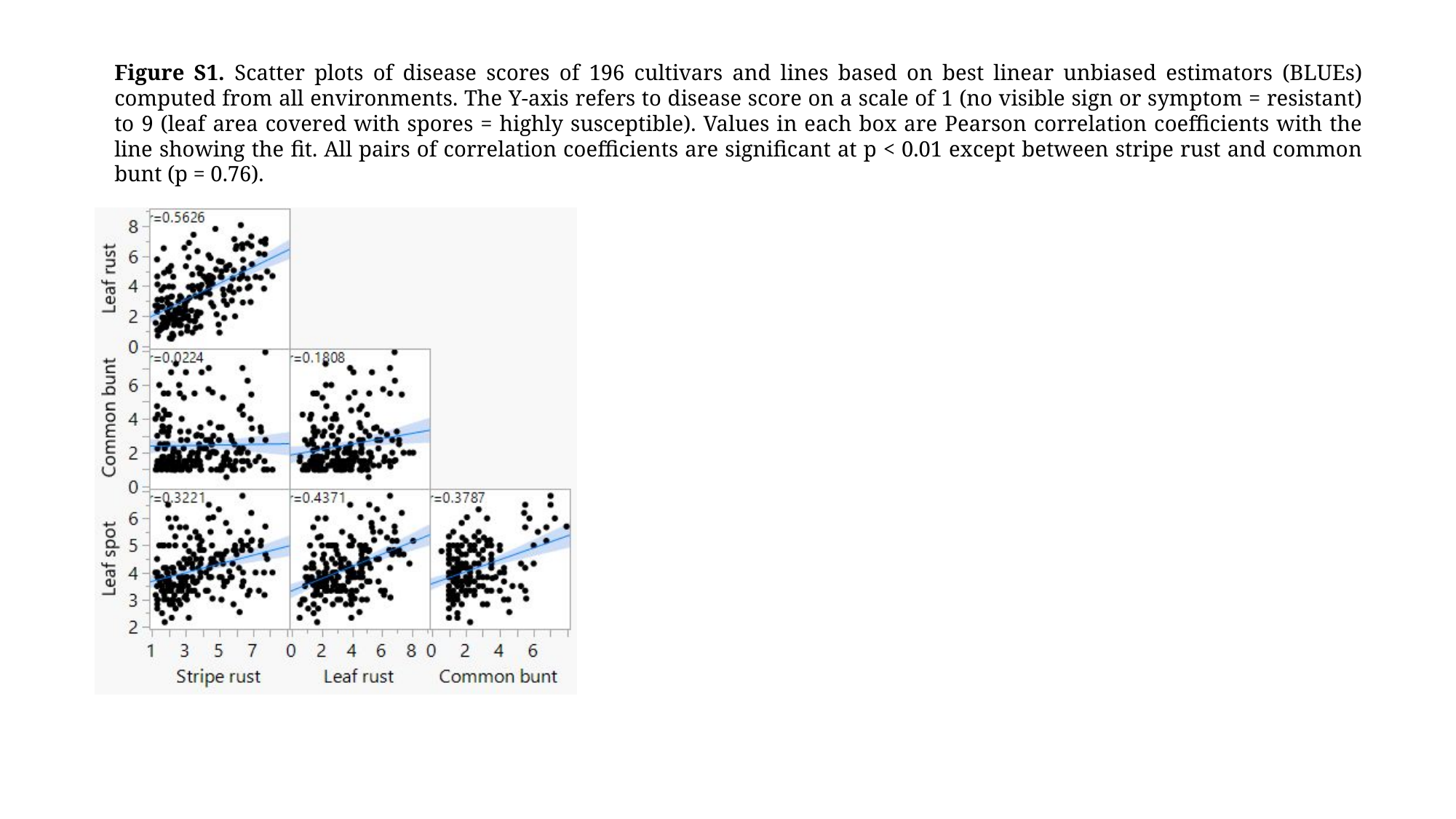

Figure S1. Scatter plots of disease scores of 196 cultivars and lines based on best linear unbiased estimators (BLUEs) computed from all environments. The Y-axis refers to disease score on a scale of 1 (no visible sign or symptom = resistant) to 9 (leaf area covered with spores = highly susceptible). Values in each box are Pearson correlation coefficients with the line showing the fit. All pairs of correlation coefficients are significant at p < 0.01 except between stripe rust and common bunt (p = 0.76).

## Slide 2
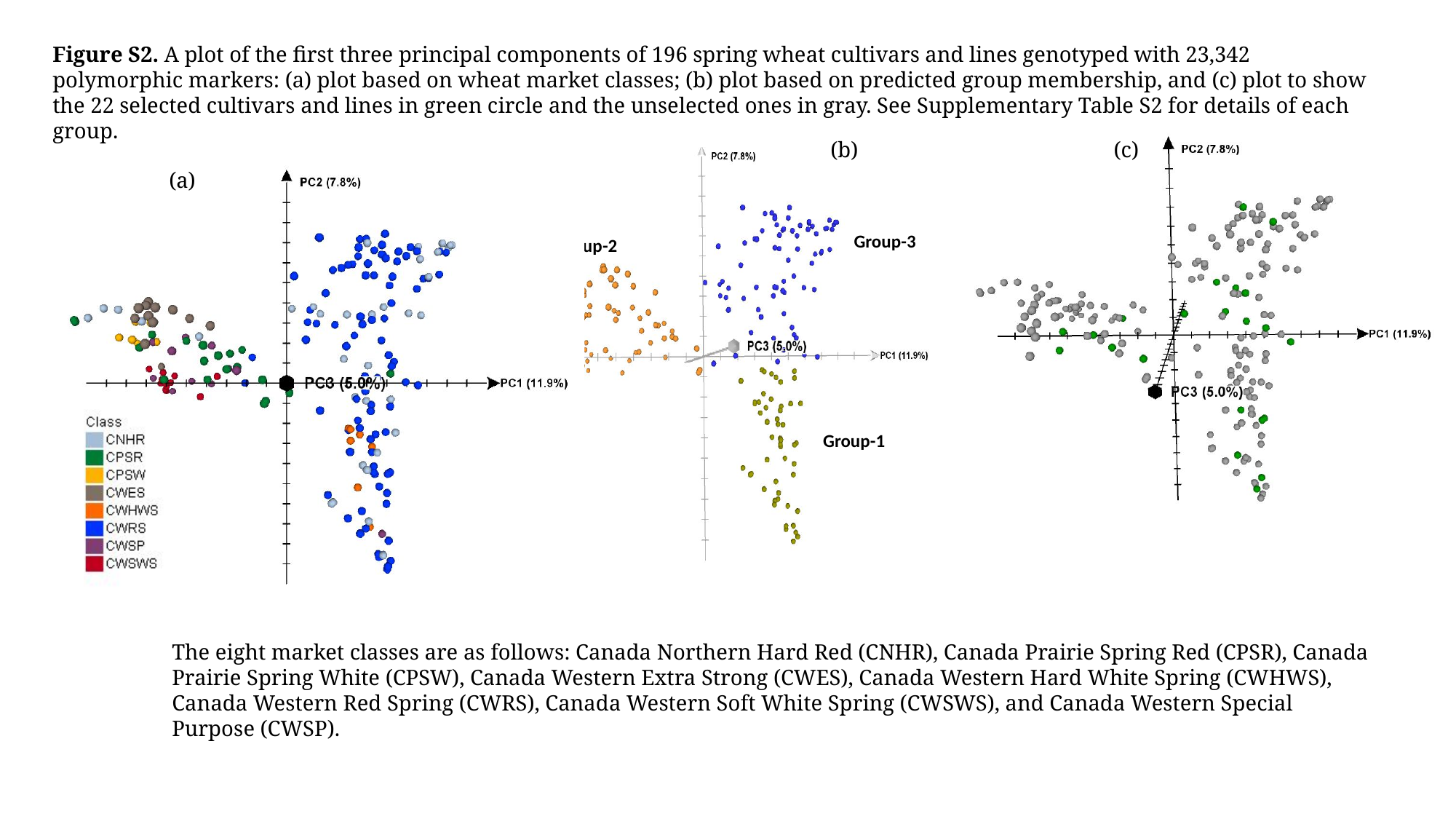

Figure S2. A plot of the first three principal components of 196 spring wheat cultivars and lines genotyped with 23,342 polymorphic markers: (a) plot based on wheat market classes; (b) plot based on predicted group membership, and (c) plot to show the 22 selected cultivars and lines in green circle and the unselected ones in gray. See Supplementary Table S2 for details of each group.
(b)
(c)
Group-1
Group-3
Group-2
(a)
The eight market classes are as follows: Canada Northern Hard Red (CNHR), Canada Prairie Spring Red (CPSR), Canada Prairie Spring White (CPSW), Canada Western Extra Strong (CWES), Canada Western Hard White Spring (CWHWS), Canada Western Red Spring (CWRS), Canada Western Soft White Spring (CWSWS), and Canada Western Special Purpose (CWSP).

## Slide 3
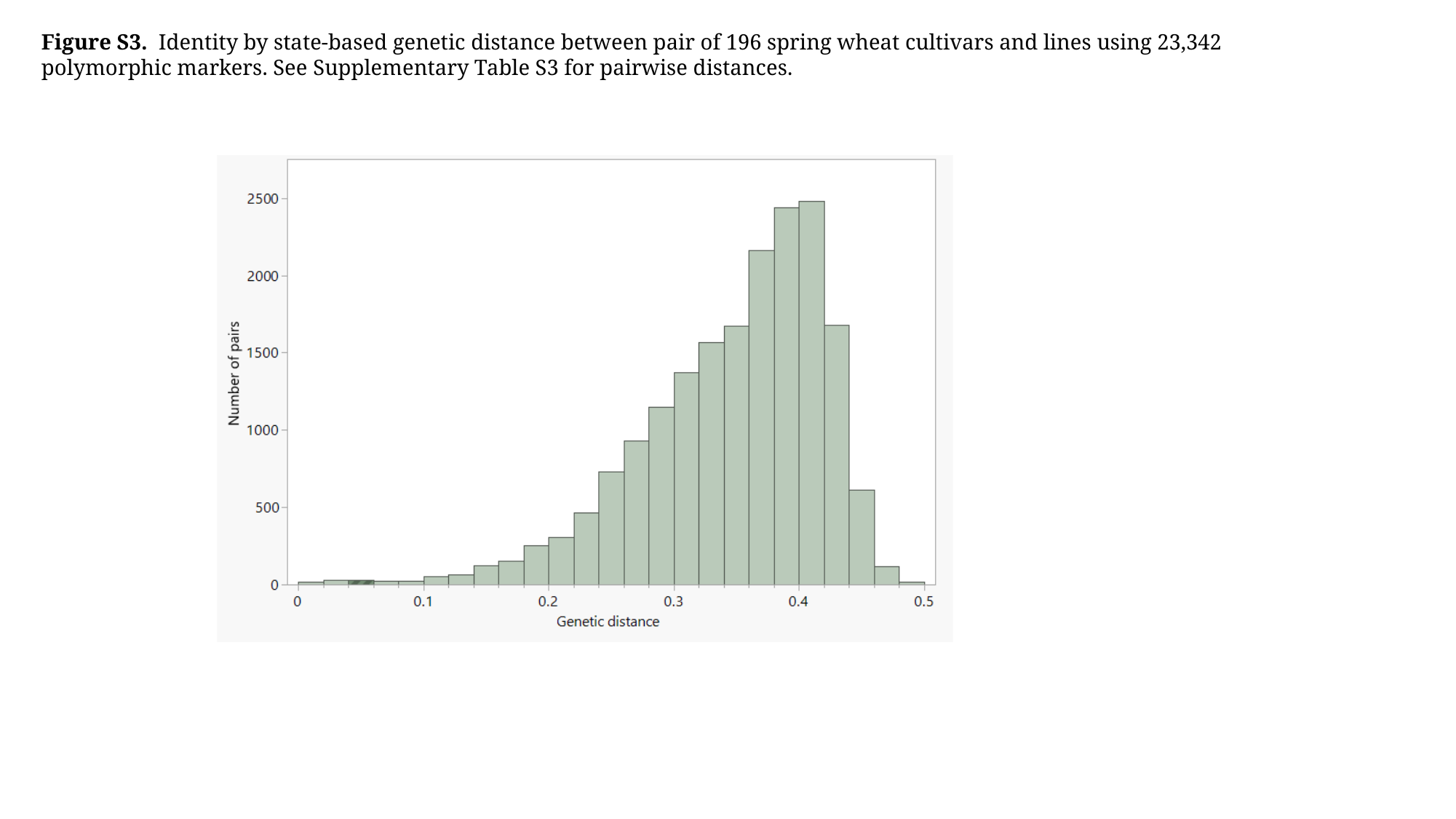

Figure S3. Identity by state-based genetic distance between pair of 196 spring wheat cultivars and lines using 23,342 polymorphic markers. See Supplementary Table S3 for pairwise distances.

## Slide 4
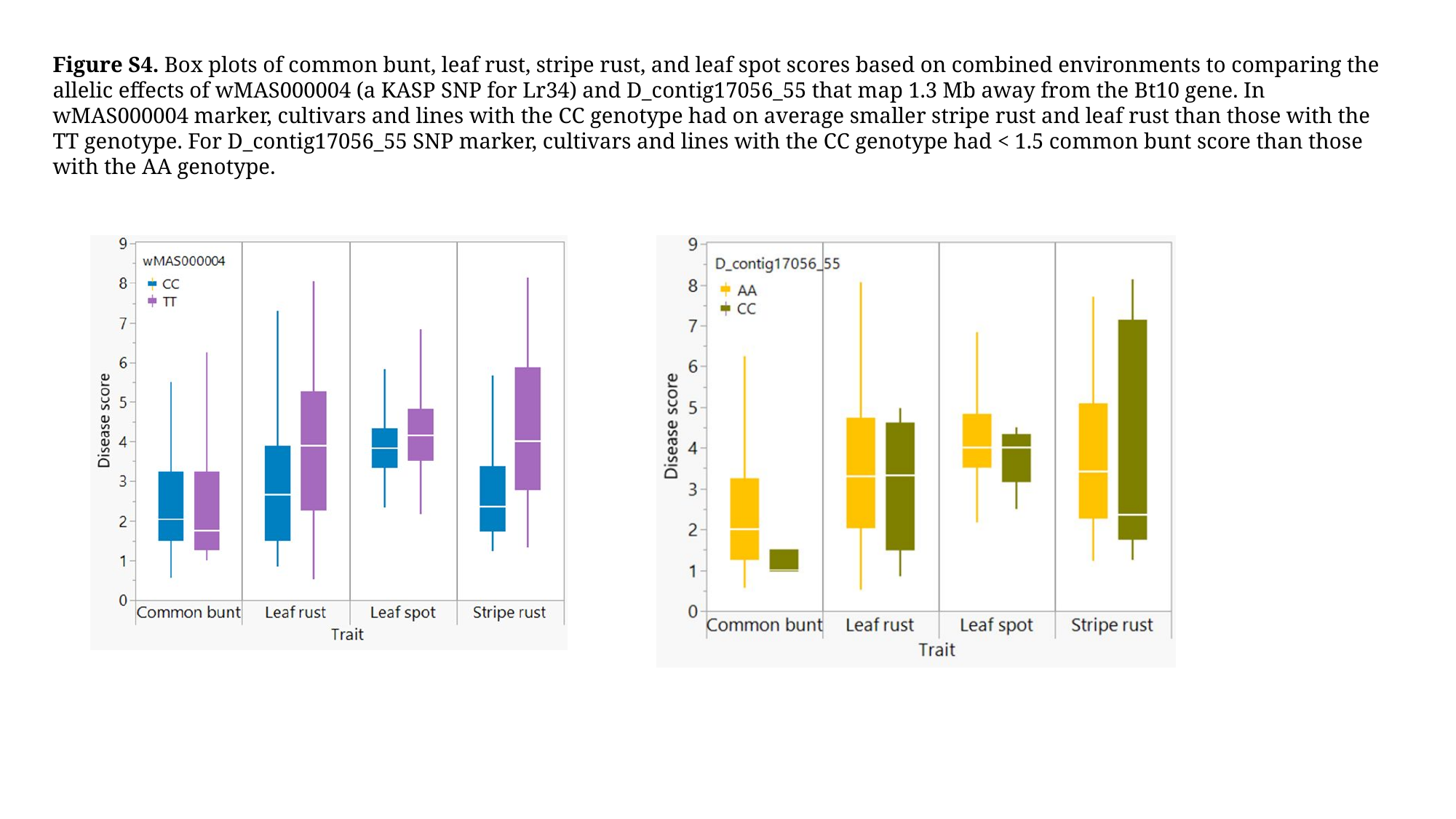

Figure S4. Box plots of common bunt, leaf rust, stripe rust, and leaf spot scores based on combined environments to comparing the allelic effects of wMAS000004 (a KASP SNP for Lr34) and D_contig17056_55 that map 1.3 Mb away from the Bt10 gene. In wMAS000004 marker, cultivars and lines with the CC genotype had on average smaller stripe rust and leaf rust than those with the TT genotype. For D_contig17056_55 SNP marker, cultivars and lines with the CC genotype had < 1.5 common bunt score than those with the AA genotype.
