# Supplementary material for: Identification of Disease Resistance Parents and Genome-Wide Association Mapping of Resistance in Spring Wheat
Source: Plants (Basel). 2022 Oct 28;11(21):2905. doi: 10.3390/plants11212905 (PMC9658635; doi:10.3390/plants11212905)
Supplement: Supplementary file 1 [file plants-11-02905-s001.zip › Table S7 ANOVA.pdf]

Table S7. Analysis of Variance (ANOVA) comparing disease severity of **selected and unselected genotypes** at each of the 37 QTLs identified using genome-wide association analysis. Disease resistant genotypes were selected based on the restricted linear phenotypic selection index (RLPSI) and the overall disease severity recorded in all combined environments.

A) Comparisons at the five stripe rust QTLs

QYr.dms-1B

Source	DF	Sum of Squares	Mean Square	F Ratio	Prob > F
Selection using RLPSI	1	82.76075	82.7608	28.8467	<0.001
Error	194	556.58312	2.8690		
Total	195	639.34388			

Comparison of mean disease severity of genotypes selected based on RLPSI (Yes) with unselected ones (No)

Selected	Number	Mean	Std Error	Lower 95%	Upper 95%
No	174	3.99943	0.12841	3.7462	4.2527
Yes	22	1.94091	0.36112	1.2287	2.6531

QYr.dms-2B

Source	DF	Sum of Squares	Mean Square	F Ratio	Prob > F
Selection using RLPSI	1	82.76075	82.7608	28.8467	<0.001
Error	194	556.58312	2.8690		
Total	195	639.34388			

Comparison of mean disease severity of genotypes selected based on RLPSI (Yes) with unselected ones (No)

Selected	Number	Mean	Std Error	Lower 95%	Upper 95%
No	174	3.99943	0.12841	3.7462	4.2527
Yes	22	1.94091	0.36112	1.2287	2.6531

QYr.dms-4B

Source	DF	Sum of Squares	Mean Square	F Ratio	Prob > F
Selection using RLPSI	1	248.2823	248.282	87.1349	<0.001
Error	586	1669.7494	2.849		
Total	587	1918.0316			

Comparison of mean disease severity of genotypes selected based on RLPSI (Yes) with unselected ones (No)

Selected	Number	Mean	Std Error	Lower 95%	Upper 95%
No	522	3.99943	0.07388	3.8543	4.1445
Yes	66	1.94091	0.20778	1.5328	2.3490

QYr.dms-5A

Source	DF	Sum of Squares	Mean Square	F Ratio	Prob > F
Selection using RLPSI	1	82.76075	82.7608	28.8467	<0.001
Error	194	556.58312	2.8690		
Total	195	639.34388			

Comparison of mean disease severity of genotypes selected based on RLPSI (Yes) with unselected ones (No)

Selected	Number	Mean	Std Error	Lower 95%	Upper 95%
No	174	3.99943	0.12841	3.7462	4.2527
Yes	22	1.94091	0.36112	1.2287	2.6531

QYr.dms-7D

Source	DF	Sum of Squares	Mean Square	F Ratio	Prob > F
Selection using RLPSI	1	82.76075	82.7608	28.8467	<0.001
Error	194	556.58312	2.8690		
Total	195	639.34388			

Comparison of mean disease severity of genotypes selected based on RLPSI (Yes) with unselected ones (No)

Selected	Number	Mean	Std Error	Lower 95%	Upper 95%
No	174	3.99943	0.12841	3.7462	4.2527
Yes	22	1.94091	0.36112	1.2287	2.6531

B) Comparisons of thirteen leaf rust QTLs

QLr.dms-1B

Source	DF	Sum of Squares	Mean Square	F Ratio	Prob > F
Selection using RLPSI	1	401.2463	401.246	153.4918	<0.001
Error	782	2044.2433	2.614		
Total	783	2445.4896			

Comparison of mean disease severity of genotypes selected based on RLPSI (Yes) with unselected ones (No)

Selected	Number	Mean	Std Error	Lower 95%	Upper 95%
No	696	3.78448	0.06129	3.6642	3.9048

Selected	Number	Mean	Std Error	Lower 95%	Upper 95%
Yes	88	1.51818	0.17235	1.1799	1.8565

QLr.dms-2A

Source	DF	Sum of Squares	Mean Square	F Ratio	Prob > F
Selection using RLPSI	1	100.31157	100.312	38.0785	<0.001
Error	194	511.06083	2.634		
Total	195	611.37240			

Comparison of mean disease severity of genotypes selected based on RLPSI (Yes) with unselected ones (No)

Selected	Number	Mean	Std Error	Lower 95%	Upper 95%
No	174	3.78448	0.12304	3.5418	4.0272
Yes	22	1.51818	0.34604	0.8357	2.2007

QLr.dms-2B.1

Source	DF	Sum of Squares	Mean Square	F Ratio	Prob > F
Selection using RLPSI	1	100.31157	100.312	38.0785	<0.001
Error	194	511.06083	2.634		
Total	195	611.37240			

Comparison of mean disease severity of genotypes selected based on RLPSI (Yes) with unselected ones (No)

Selected	Number	Mean	Std Error	Lower 95%	Upper 95%
No	174	3.78448	0.12304	3.5418	4.0272
Yes	22	1.51818	0.34604	0.8357	2.2007

QLr.dms-2B.2

Source	DF	Sum of Squares	Mean Square	F Ratio	Prob > F
Selection using RLPSI	1	1003.1157	1003.12	384.3183	<0.001
Error	1958	5110.6083	2.61		
Total	1959	6113.7240			

Comparison of mean disease severity of genotypes selected based on RLPSI (Yes) with unselected ones (No)

Selected	Number	Mean	Std Error	Lower 95%	Upper 95%
No	1740	3.78448	0.03873	3.7085	3.8604
Yes	220	1.51818	0.10892	1.3046	1.7318

QLr.dms-2D

Source	DF	Sum of Squares	Mean Square	F Ratio	Prob > F
Selection using RLPSI	1	501.5578	501.558	191.9629	<0.001
Error	978	2555.3042	2.613		
Total	979	3056.8620			

Comparison of mean disease severity of genotypes selected based on RLPSI (Yes) with unselected ones (No)

Selected	Number	Mean	Std Error	Lower 95%	Upper 95%
No	870	3.78448	0.05480	3.6769	3.8920
Yes	110	1.51818	0.15412	1.2157	1.8206

QLr.dms-3A

Source	DF	Sum of Squares	Mean Square	F Ratio	Prob > F
Selection using RLPSI	1	100.31157	100.312	38.0785	<0.001
Error	194	511.06083	2.634		
Total	195	611.37240			

Comparison of mean disease severity of genotypes selected based on RLPSI (Yes) with unselected ones (No)

Selected	Number	Mean	Std Error	Lower 95%	Upper 95%
No	174	3.78448	0.12304	3.5418	4.0272
Yes	22	1.51818	0.34604	0.8357	2.2007

QLr.dms-3B.1

Source	DF	Sum of Squares	Mean Square	F Ratio	Prob > F
Selection using RLPSI	1	100.31157	100.312	38.0785	<0.001
Error	194	511.06083	2.634		
Total	195	611.37240			

Comparison of mean disease severity of genotypes selected based on RLPSI (Yes) with unselected ones (No)

Selected	Number	Mean	Std Error	Lower 95%	Upper 95%
No	174	3.78448	0.12304	3.5418	4.0272
Yes	22	1.51818	0.34604	0.8357	2.2007

QLr.dms-3B.2

Source	DF	Sum of Squares	Mean Square	F Ratio	Prob > F
Selection using RLPSI	1	401.2463	401.246	153.4918	<0.001
Error	782	2044.2433	2.614		
Total	783	2445.4896			

Comparison of mean disease severity of genotypes selected based on RLPSI (Yes) with unselected ones (No)

Selected	Number	Mean	Std Error	Lower 95%	Upper 95%
No	696	3.78448	0.06129	3.6642	3.9048
Yes	88	1.51818	0.17235	1.1799	1.8565

QLr.dms-3D

Source	DF	Sum of Squares	Mean Square	F Ratio	Prob > F
Selection using RLPSI	1	100.31157	100.312	38.0785	<0.001
Error	194	511.06083	2.634		
Total	195	611.37240			

Comparison of mean disease severity of genotypes selected based on RLPSI (Yes) with unselected ones (No)

Selected	Number	Mean	Std Error	Lower 95%	Upper 95%
No	174	3.78448	0.12304	3.5418	4.0272
Yes	22	1.51818	0.34604	0.8357	2.2007

QLr.dms-5A.1

Source	DF	Sum of Squares	Mean Square	F Ratio	Prob > F
Selection using RLPSI	1	200.6231	200.623	76.5496	<0.001
Error	390	1022.1217	2.621		
Total	391	1222.7448			

Comparison of mean disease severity of genotypes selected based on RLPSI (Yes) with unselected ones (No)

Selected	Number	Mean	Std Error	Lower 95%	Upper 95%
No	348	3.78448	0.08678	3.6139	3.9551
Yes	44	1.51818	0.24406	1.0383	1.9980

QLr.dms-5A.2

Source	DF	Sum of Squares	Mean Square	F Ratio	Prob > F
Selection using RLPSI	1	100.31157	100.312	38.0785	<0.001
Error	194	511.06083	2.634		
Total	195	611.37240			

Comparison of mean disease severity of genotypes selected based on RLPSI (Yes) with unselected ones (No)

Selected	Number	Mean	Std Error	Lower 95%	Upper 95%
No	174	3.78448	0.12304	3.5418	4.0272
Yes	22	1.51818	0.34604	0.8357	2.2007

QLr.dms-5B

Source	DF	Sum of Squares	Mean Square	F Ratio	Prob > F
Selection using RLPSI	1	200.6231	200.623	76.5496	<0.001
Error	390	1022.1217	2.621		
Total	391	1222.7448			

Comparison of mean disease severity of genotypes selected based on RLPSI (Yes) with unselected ones (No)

Selected	Number	Mean	Std Error	Lower 95%	Upper 95%
No	348	3.78448	0.08678	3.6139	3.9551
Yes	44	1.51818	0.24406	1.0383	1.9980

QLr.dms-7B

Source	DF	Sum of Squares	Mean Square	F Ratio	Prob > F
Selection using RLPSI	1	100.31157	100.312	38.0785	<0.001
Error	194	511.06083	2.634		
Total	195	611.37240			

Comparison of mean disease severity of genotypes selected based on RLPSI (Yes) with unselected ones (No)

Selected	Number	Mean	Std Error	Lower 95%	Upper 95%
No	174	3.78448	0.12304	3.5418	4.0272
Yes	22	1.51818	0.34604	0.8357	2.2007

C) Comparisons of seven leaf spot QTLs

QLs.dms-1B

Source	DF	Sum of Squares	Mean Square	F Ratio	Prob > F
Selection using RLPSI	1	21.39344	21.3934	28.9616	<0.001
Error	194	143.30432	0.7387		
Total	195	164.69776			

Comparison of mean disease severity of genotypes selected based on RLPSI (Yes) with unselected ones (No)

Selected	Number	Mean	Std Error	Lower 95%	Upper 95%
No	174	4.25115	0.06516	4.1226	4.3797
Yes	22	3.20455	0.18324	2.8431	3.5659

QLs.dms-2A.1

Source	DF	Sum of Squares	Mean Square	F Ratio	Prob > F
Selection using RLPSI	1	21.39344	21.3934	28.9616	<0.001
Error	194	143.30432	0.7387		
Total	195	164.69776			

Comparison of mean disease severity of genotypes selected based on RLPSI (Yes) with unselected ones (No)

Selected	Number	Mean	Std Error	Lower 95%	Upper 95%
No	174	4.25115	0.06516	4.1226	4.3797
Yes	22	3.20455	0.18324	2.8431	3.5659

QLs.dms-2A.2

Source	DF	Sum of Squares	Mean Square	F Ratio	Prob > F
Selection using RLPSI	1	128.36064	128.361	175.2627	<0.001
Error	1174	859.82589	0.732		
Total	1175	988.18653			

Comparison of mean disease severity of genotypes selected based on RLPSI (Yes) with unselected ones (No)

Selected	Number	Mean	Std Error	Lower 95%	Upper 95%
No	1044	4.25115	0.02649	4.1992	4.3031
Yes	132	3.20455	0.07449	3.0584	3.3507

QLs.dms-2B

Source	DF	Sum of Squares	Mean Square	F Ratio	Prob > F
Selection using RLPSI	1	21.39344	21.3934	28.9616	<0.001
Error	194	143.30432	0.7387		
Total	195	164.69776			

Comparison of mean disease severity of genotypes selected based on RLPSI (Yes) with unselected ones (No)

Selected	Number	Mean	Std Error	Lower 95%	Upper 95%
No	174	4.25115	0.06516	4.1226	4.3797
Yes	22	3.20455	0.18324	2.8431	3.5659

QLs.dms-3B

Source	DF	Sum of Squares	Mean Square	F Ratio	Prob > F
Selection using RLPSI	1	21.39344	21.3934	28.9616	<0.001
Error	194	143.30432	0.7387		
Total	195	164.69776			

Comparison of mean disease severity of genotypes selected based on RLPSI (Yes) with unselected ones (No)

Selected	Number	Mean	Std Error	Lower 95%	Upper 95%
No	174	4.25115	0.06516	4.1226	4.3797
Yes	22	3.20455	0.18324	2.8431	3.5659

QLs.dms-4A

Source	DF	Sum of Squares	Mean Square	F Ratio	Prob > F
Selection using RLPSI	1	21.39344	21.3934	28.9616	<0.001
Error	194	143.30432	0.7387		
Total	195	164.69776			

Comparison of mean disease severity of genotypes selected based on RLPSI (Yes) with unselected ones (No)

Selected	Number	Mean	Std Error	Lower 95%	Upper 95%
No	174	4.25115	0.06516	4.1226	4.3797
Yes	22	3.20455	0.18324	2.8431	3.5659

QLs.dms-7D

Source	DF	Sum of Squares	Mean Square	F Ratio	Prob > F
Selection using RLPSI	1	21.39344	21.3934	28.9616	<0.001
Error	194	143.30432	0.7387		
Total	195	164.69776			

Comparison of mean disease severity of genotypes selected based on RLPSI (Yes) with unselected ones (No)

Selected	Number	Mean	Std Error	Lower 95%	Upper 95%
No	174	4.25115	0.06516	4.1226	4.3797
Yes	22	3.20455	0.18324	2.8431	3.5659

D) Comparisons of twelve common bunt QTLs

QCbt.dms-1A.1

Source	DF	Sum of Squares	Mean Square	F Ratio	Prob > F
Selection using RLPSI	1	27.98955	27.9895	11.4419	<0.001
Error	194	474.56856	2.4462		
Total	195	502.55811			

Comparison of mean disease severity of genotypes selected based on RLPSI (Yes) with unselected ones (No)

Selected	Number	Mean	Std Error	Lower 95%	Upper 95%
No	174	2.64713	0.11857	2.4133	2.8810
Yes	22	1.45000	0.33346	0.7923	2.1077

QCbt.dms-1A.2

Source	DF	Sum of Squares	Mean Square	F Ratio	Prob > F
Selection using RLPSI	1	55.9791	55.9791	23.0018	<0.001
Error	390	949.1371	2.4337		
Total	391	1005.1162			

Comparison of mean disease severity of genotypes selected based on RLPSI (Yes) with unselected ones (No)

Selected	Number	Mean	Std Error	Lower 95%	Upper 95%
No	348	2.64713	0.08363	2.4827	2.8115
Yes	44	1.45000	0.23518	0.9876	1.9124

QCbt.dms-1A.3

Source	DF	Sum of Squares	Mean Square	F Ratio	Prob > F
Selection using RLPSI	1	27.98955	27.9895	11.4419	<0.001
Error	194	474.56856	2.4462		
Total	195	502.55811			

Comparison of mean disease severity of genotypes selected based on RLPSI (Yes) with unselected ones (No)

Selected	Number	Mean	Std Error	Lower 95%	Upper 95%
No	174	2.64713	0.11857	2.4133	2.8810
Yes	22	1.45000	0.33346	0.7923	2.1077

QCbt.dms-1B

Source	DF	Sum of Squares	Mean Square	F Ratio	Prob > F
Selection using RLPSI	1	27.98955	27.9895	11.4419	<0.001
Error	194	474.56856	2.4462		

Source	DF	Sum of Squares	Mean Square	F Ratio	Prob > F
Total	195	502.55811			

Comparison of mean disease severity of genotypes selected based on RLPSI (Yes) with unselected ones (No)

Selected	Number	Mean	Std Error	Lower 95%	Upper 95%
No	174	2.64713	0.11857	2.4133	2.8810
Yes	22	1.45000	0.33346	0.7923	2.1077

QCbt.dms-1D

Source	DF	Sum of Squares	Mean Square	F Ratio	Prob > F
Selection using RLPSI	1	27.98955	27.9895	11.4419	<0.001
Error	194	474.56856	2.4462		
Total	195	502.55811			

Comparison of mean disease severity of genotypes selected based on RLPSI (Yes) with unselected ones (No)

Selected	Number	Mean	Std Error	Lower 95%	Upper 95%
No	174	2.64713	0.11857	2.4133	2.8810
Yes	22	1.45000	0.33346	0.7923	2.1077

QCbt.dms-2B

Source	DF	Sum of Squares	Mean Square	F Ratio	Prob > F
Selection using RLPSI	1	27.98955	27.9895	11.4419	<0.001
Error	194	474.56856	2.4462		
Total	195	502.55811			

Comparison of mean disease severity of genotypes selected based on RLPSI (Yes) with unselected ones (No)

Selected	Number	Mean	Std Error	Lower 95%	Upper 95%
No	174	2.64713	0.11857	2.4133	2.8810
Yes	22	1.45000	0.33346	0.7923	2.1077

QCbt.dms-3A.1

Source	DF	Sum of Squares	Mean Square	F Ratio	Prob > F
Selection using RLPSI	1	27.98955	27.9895	11.4419	<0.001
Error	194	474.56856	2.4462		
Total	195	502.55811			

Comparison of mean disease severity of genotypes selected based on RLPSI (Yes) with unselected ones (No)

Selected	Number	Mean	Std Error	Lower 95%	Upper 95%
No	174	2.64713	0.11857	2.4133	2.8810
Yes	22	1.45000	0.33346	0.7923	2.1077

QCbt.dms-3A.2

Source	DF	Sum of Squares	Mean Square	F Ratio	Prob > F
Selection using RLPSI	1	27.98955	27.9895	11.4419	<0.001
Error	194	474.56856	2.4462		
Total	195	502.55811			

Comparison of mean disease severity of genotypes selected based on RLPSI (Yes) with unselected ones (No)

Selected	Number	Mean	Std Error	Lower 95%	Upper 95%
No	174	2.64713	0.11857	2.4133	2.8810
Yes	22	1.45000	0.33346	0.7923	2.1077

QCbt.dms-5D.1

Source	DF	Sum of Squares	Mean Square	F Ratio	Prob > F
Selection using RLPSI	1	27.98955	27.9895	11.4419	<0.001
Error	194	474.56856	2.4462		
Total	195	502.55811			

Comparison of mean disease severity of genotypes selected based on RLPSI (Yes) with unselected ones (No)

Selected	Number	Mean	Std Error	Lower 95%	Upper 95%
No	174	2.64713	0.11857	2.4133	2.8810
Yes	22	1.45000	0.33346	0.7923	2.1077

QCbt.dms-5D.2

Source	DF	Sum of Squares	Mean Square	F Ratio	Prob > F
Selection using RLPSI	1	27.98955	27.9895	11.4419	<0.001
Error	194	474.56856	2.4462		
Total	195	502.55811			

Comparison of mean disease severity of genotypes selected based on RLPSI (Yes) with unselected ones (No)

Selected	Number	Mean	Std Error	Lower 95%	Upper 95%
No	174	2.64713	0.11857	2.4133	2.8810

Selected	Number	Mean	Std Error	Lower 95%	Upper 95%
Yes	22	1.45000	0.33346	0.7923	2.1077

QCbt.dms-6D

Source	DF	Sum of Squares	Mean Square	F Ratio	Prob > F
Selection using RLPSI	1	27.98955	27.9895	11.4419	<0.001
Error	194	474.56856	2.4462		
Total	195	502.55811			

Comparison of mean disease severity of genotypes selected based on RLPSI (Yes) with unselected ones (No)

Selected	Number	Mean	Std Error	Lower 95%	Upper 95%
No	174	2.64713	0.11857	2.4133	2.8810
Yes	22	1.45000	0.33346	0.7923	2.1077

QCbt.dms-7A

Source	DF	Sum of Squares	Mean Square	F Ratio	Prob > F
Selection using RLPSI	1	27.98955	27.9895	11.4419	<0.001
Error	194	474.56856	2.4462		
Total	195	502.55811			

Comparison of mean disease severity of genotypes selected based on RLPSI (Yes) with unselected ones (No)

Selected	Number	Mean	Std Error	Lower 95%	Upper 95%
No	174	2.64713	0.11857	2.4133	2.8810
Yes	22	1.45000	0.33346	0.7923	2.1077
