# Supplementary material for: Identification of Disease Resistance Parents and Genome-Wide Association Mapping of Resistance in Spring Wheat
Source: Plants (Basel). 2022 Oct 28;11(21):2905. doi: 10.3390/plants11212905 (PMC9658635; doi:10.3390/plants11212905)
Supplement: Supplementary file 1 [file plants-11-02905-s001.zip › Table S8 RLPSI code.pdf]

Table S8. The Kempthorne and Nordskog Restrictive Linear Phenotypic Selection Index (RLPSI) code in R to compute the different selection parameters.

```
#Economic weights per trait: Stripe rust, leaf rust, leaf spot, and common bunt (-1, -3, -5, -7, and -9 for each); grain yield (1, 3, 5, 7, and 9)

#Restricted (constrained trait): expected genetic gain for grain yield = 0

# The economic weights for the example below are as follows:  stripe rust = -7, leaf rust = -9, leaf spot = -9, common bunt = -9, grain yield =1

# Restrictions: 0= no restriction; 1 = expected genetic gain restricted (fixed) to zero

# RLPSI analysis was done by restricting the expected genetic gain for grain yield to zero

# This code below is an example for just one of the 3,125 economic weights used in our study.


rm(list=ls())

setwd("C:/R-packages/RLPSI/Data")


P<- as.matrix(read.csv("POP1-P-matrix.csv", header = TRUE, nrows =5))

G <- as.matrix(read.csv("POP1-G-Matrix.csv", header = TRUE, nrows =5))

Y<- as.matrix(read.csv("POP1_Data_Non-cenetered.csv", header = TRUE, nrows =196))

GID<- as.matrix(read.csv("POP1_GID.csv", header = TRUE, nrows =196))


IP=solve(P);IP                    # P inverse matrix

w=c(-7,-9,-9,-9,1); w        # Economic weights for stripe rust, leaf rust, leaf spot, common bunt, and grain yield, respectively.

b=IP%*%G%*%w; b                   # LPSI vector of coefficients


CC1 <- c(0,0,0,0,1)          # Matrix of restrictions for stripe rust, leaf rust, leaf spot, common bunt, and grain yield, respectively.


Ut=rbind(CC1);   Ut

At=Ut%*%G; At
```

```
II=diag(c(1,1,1,1,1)); II

IQ=solve(At%*%IP%*%t(At)); IQ

Q=IP%*%t(At)%*%IQ%*%At; Q

KK=II-Q; KK

bs=KK%*%b; bs                    # RLPSI vector of coefficients


ks=1.755                         # Selecion intensity

Vp=t(bs)%*%P%*%bs; Vp ; SD=sqrt(Vp); SD        # Variance and standard deviation of the RLPSI

Es=(ks*(G%*%bs))/as.vector(SD) ; Es             # Estimated Expected genetic gain per trait

Rs=ks*SD;Rs                              # Estimated LRPSI Selection response

VH=t(w)%*%G%*%w; SH=sqrt(VH);  VH; SH             # Variance and standard deviation of the net
genetic merit H

corHI=SD/SH;   corHI                     # Estimated correlation between the RLPSI and H


LSI=Y%*%bs                               # Estimated RLPSI values

datos <-data.frame(GID=GID, Y=Y,LSI=LSI)

datos <- datos[with(datos, order(-LSI)), ]          # Orden inverso


write.table(datos, "C:/R-packages/RLPSI/Results-RLPSI-Weight_1-all.txt", sep="\t")

X5=datos[1:20,1:7]

write.table(X5, "C:/R-packages/RLPSI/Results-RLPSI-Weight_1-selected.txt", sep="\t")
```
